# An Efficient Optimization Method for Large-Solution Space Electromagnetic Automatic Design

**DOI:** 10.3390/ma18051159

**Published:** 2025-03-05

**Authors:** Lingyan He, Fengling Peng, Xing Chen

**Affiliations:** 1 Department of Industrial Engineering, Pittsburgh Institute, Sichuan University, Chengdu 610041, China; helingyan@stu.scu.edu.cn; 2 Department of Information and Communication Engineering, College of Electronics and Information Engineering, Sichuan University, Chengdu 610041, China; pengfenglingtg@126.com

**Keywords:** crossover probability, reinforcement learning, automatic design

## Abstract

In the field of electromagnetic design, it is sometimes necessary to search for the optimal design solution (i.e., the optimal solution) within a large solution space to complete the optimization. However, traditional optimization methods are not only slow in searching for the solution space but are also prone to becoming trapped in local optima, leading to optimization failure. This paper proposes a dual-population genetic algorithm to quickly find the optimal solution for electromagnetic optimization problems in large solution spaces. The method involves two populations: the first population uses the powerful dynamic decision-making ability of reinforcement learning to adjust the crossover probability, making the optimization process more stable and enhancing the global optimization capability of the algorithm. The second population accelerates the convergence speed of the algorithm by employing a “leader dominance” mechanism, allowing the population to quickly approach the optimal solution. The two populations are integrated through an immigration operator, improving optimization efficiency. The effectiveness of the proposed method is demonstrated through the optimization design of an electromagnetic metasurface material. Furthermore, the method designed in this paper is not limited to the electromagnetic field and has practical value in other engineering optimization areas, such as vehicle routing optimization, energy system optimization, and fluid dynamics optimization, etc.

## 1. Introduction

**Figure 1:**
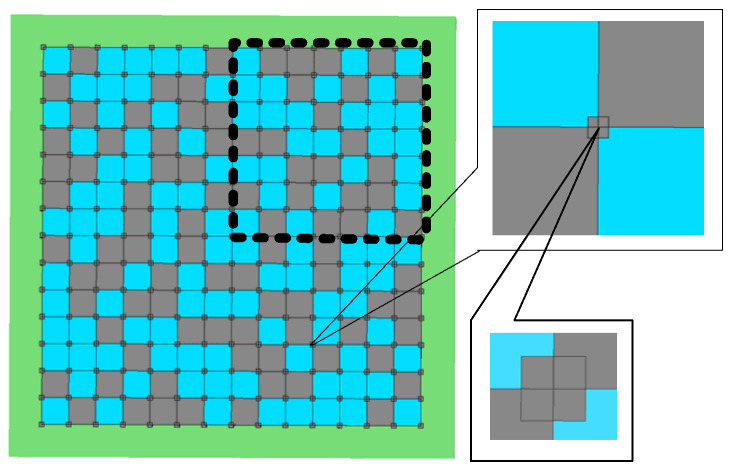
Random lattice electromagnetic super-surface.

With the development of industrialization in society, various electromagnetic (EM) devices have been designed and applied across multiple fields [1,2,3]. Designing EM devices typically requires theoretical modeling followed by optimization of the design variables. However, due to the complexity of EM theory, designers sometimes need to find optimal solutions within a vast solution space. The size of this solution space is generally related to the number of design variables and their range of values, for instance, designing a random EM metasurface (an application case of the algorithm proposed in this paper) as shown in Figure 1. With each square having two possible materials, even optimizing a 7 × 7 structure results in a solution space size of 2^49^ = 562,949,953,421,312. Generally, when the size of the solution space exceeds one trillion, it is considered a large solution space (LSS) problem. Traditional manual optimization methods become extremely difficult to find the optimal solution at this scale. Hence, the need for automatic EM optimization methods arises.

Such automatic optimization methods utilize algorithms (such as genetic algorithms (GA), particle swarm optimization, etc.) to automatically find the optimal solution for a problem [4,5,6]. A commonly used optimization method involves generating multiple solutions in the solution space at once to form a population. Then, numerical simulation tools (such as HFSS or CST) are used to evaluate each solution. Based on the evaluation results, a new population is generated, and this process is repeated until the optimal solution is found [7]. Although the effectiveness of this method has been proven, the optimization process may require hundreds to tens of thousands of numerical simulations, leading to expensive computational costs.

To address this issue, methods based on surrogate models have been introduced [8,9,10,11]. The core idea of this method is to use machine learning algorithms to create a mathematical model (i.e., a surrogate model) to replace the time-consuming numerical simulations, thus, enabling rapid evaluation of solutions. However, creating a surrogate model requires sampling, and acquiring these samples necessitates multiple numerical simulations. Therefore, the number of samples required in the early stages is often difficult to determine. While collecting more samples can improve the prediction accuracy of the surrogate model, it is also very time-consuming.

To resolve the conflict between the computational overhead of collecting samples and the evaluation accuracy of surrogate models, methods based on self-growing surrogate models have emerged [12,13]. The major advantage of these methods is that they allow designers to avoid excessive worry about the initial sample size. Initially, only a few samples are collected to train a rough surrogate model. Then, as the algorithm iterates and generates new samples, the surrogate model is retrained, allowing it to grow along with the iterations of the algorithm and gradually improve its evaluation accuracy. Some scholars have proposed constructing multiple local surrogates to reduce the requirement for samples [14]. This method creates different local surrogate models based on the distinct characteristics of the solutions. Each surrogate model is responsible for predicting the quality of a specific solution, without needing to account for the entire solution space. As a result, this approach can further improve the prediction accuracy of the surrogate model without increasing the number of samples.

Since constructing surrogate models requires collecting samples, and sample collection still involves repeatedly calling time-consuming numerical simulation tools, this can significantly reduce optimization efficiency. Moreover, because surrogate models have lower evaluation accuracy than numerical simulations, they can easily misguide the optimization direction. Based on this, this paper does not propose improvements from the perspective of surrogate models but instead improves the optimization efficiency of large-solution space problems by enhancing the performance of the optimization algorithm itself (i.e., the genetic algorithm designed in this paper). Furthermore, the proposed algorithm is not limited to electromagnetic optimization design but can also be applied to efficiently solve combinatorial optimization problems in other engineering fields.

The main contributions of this paper are the following:

1. A population-based method based on reinforcement learning is proposed to achieve more stable exploration of large solution spaces, thereby enhancing the global search capability of the optimization algorithm and avoiding local optima.
2. A leadership-dominant mechanism for the population is introduced, where the optimal solution is mimicked multiple times by other solutions in the population. This improves the exploration speed of large solution spaces and reduces the number of iterations.
3. By combining the above-two points, an efficient optimization method is designed and implemented to address large-solution space optimization problems in the electromagnetic field. Additionally, this method is applicable to other engineering fields and demonstrates certain general applicability.

## 2. Advantages of Double Population Algorithm

Traditional optimization algorithms are almost all single-population algorithms. The biggest disadvantage of these methods is that it is difficult to balance both exploration and exploitation simultaneously. When the algorithm focuses on exploration, it needs to expand the search space as much as possible, which inevitably slows down the convergence speed. When the algorithm focuses on exploitation, it concentrates on specific local regions, which may lead to becoming stuck in local optima. In contrast, the dual-population algorithm allows the first population to focus on exploration and the second population to focus on exploitation, with mutual interaction between the two populations, thus, achieving a balance between exploration and exploitation [15,16,17]. Based on this, the paper designs an improved dual-population genetic algorithm (IDPGA) to address the large solution space optimization problem in the electromagnetic field. According to the author’s research, the application of dual-population algorithms to solve electromagnetic optimization problems is quite rare [18,19]. Furthermore, since IDPGA is itself a genetic algorithm, its applicability is not limited to the electromagnetic field; it can also be used for efficient optimization in other industrial fields involving combinatorial optimization problems.

## 3. Process Design of IDPGA

**Figure 2:**
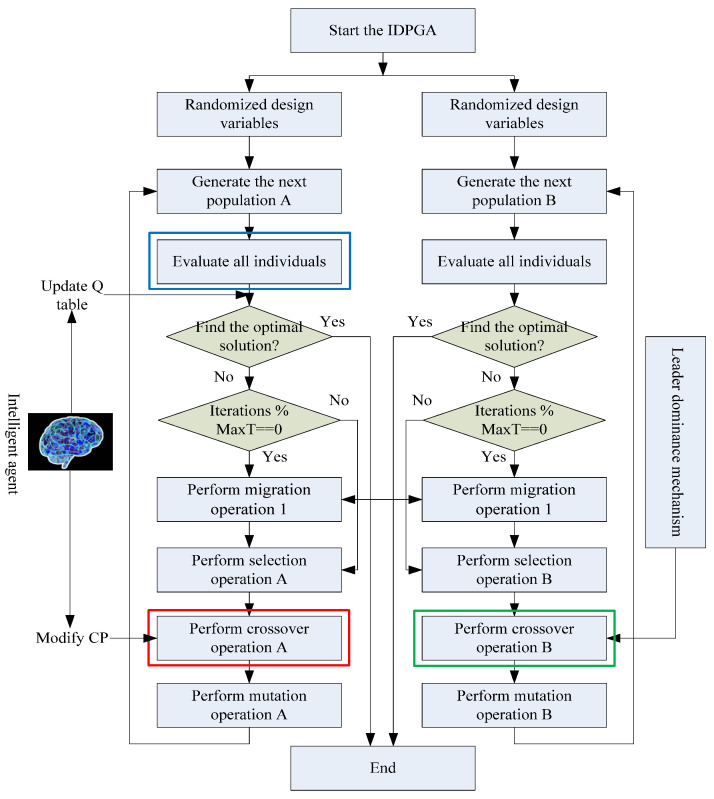
Process design of IDPGA.

The process of the IDPGA is illustrated in Figure 2. Initially, the algorithm randomly generates a set of EM solutions. These schemes are then randomly split into two equal parts, forming populations A and B. Subsequently, the EM solutions of both populations are evaluated for quality through simulation calls. Once all EM schemes are evaluated, if the optimal solution is found, the algorithm terminates. Otherwise, the iteration count *n* increases by one. If *n* is a multiple of a predetermined maximum number of iterations, *MaxT*, an immigration operator is triggered. If not, populations A and B proceed to execute their respective selection, crossover, and mutation operators to generate the next population.

In population A, a reinforcement learning algorithm (specifically Q-Learning) is used, where an intelligent agent adjusts the crossover probability (CP). The agent follows the standard Q-Learning rule, which proceeds as follows: (1) The agent performs an action to modify the CP based on the current population state; (2) After the algorithm evaluates the solutions in the population, it provides the agent with a reward score; (3) The agent records the reward score in the Q-table and, based on the Q-table, performs another action to modify the CP; (4) This process is repeated until the Q-table converges. Throughout this process, the agent adjusts the CP based on past, present, and future experiences, thereby stabilizing the evolutionary mechanism of population A. In Population B, a leadership dominance mechanism is employed during the crossover operation. This strategy involves conducting crossover operations primarily between ordinary individuals and the current best individual in Population B. Finally, an improved immigration operator is used to merge the advantages of both populations. The immigration operator is primarily used to facilitate information exchange between the two populations. Its function is equivalent to exchanging useful information between them, thereby enabling a more efficient search. The technical details of the immigration operator will be elaborated in the next section.

## 4. Design of Migration Operator

### 4.1. Analysis of Traditional Immigration Operator

**Figure 3:**
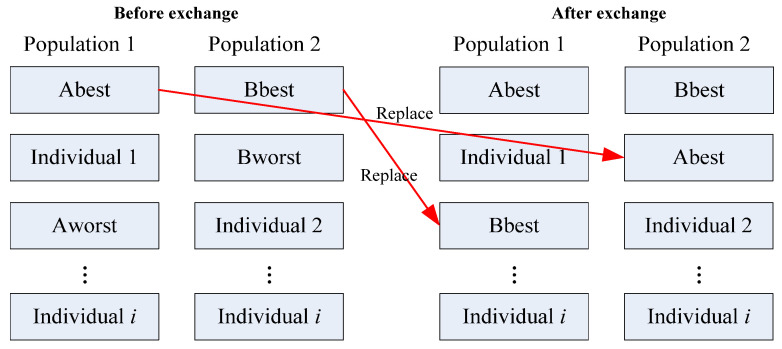
Traditional immigration operator.

Since the optimization algorithm designed in this paper aims to solve a maximization problem, the higher the fitness value of an individual (i.e., a solution), the better the quality of that individual. The traditional immigration operator first identifies individuals with the poorest fitness value (referred to as Aworst and Bworst) in their respective populations. Then, they replace these poorest fit value individuals with copies of the highest fitness value individuals (referred to as Abest and Bbest) from their own populations, as shown in Figure 3. This means replacing Bworst with a copy of Abest, and vice versa, replacing Aworst with a copy of Bbest. The major drawback of this method is its tendency to disrupt the structure of each population. Consequently, an improved immigration operator is proposed.

### 4.2. Design of Improved Immigration Operator

1. Immigration operator 1: This immigration operator differs from the traditional method in that, when exchanging the best individuals between populations, the individuals removed are not the worst but those most similar to the best individual of the opposite population. This approach ensures the exchange of high-quality genes while avoiding disruption to the population structure. To identify individuals with the highest similarity, it is necessary to calculate the similarity between two individuals, as shown in Equation (1). Here, G(i, j) represents the value of the jth design variable for the ith individual in the current population, while Gbest(j) is the value of the jth design variable for the best individual in the other population, and *N* is the number of design variables.

(1) $$\mathrm{Similar}\left(i\right)=\sum_{j=1}^{j=N}\left|G\left(i,j\right)-\mathrm{Gbest}\left(j\right)\right|/N$$

**Figure 4:**
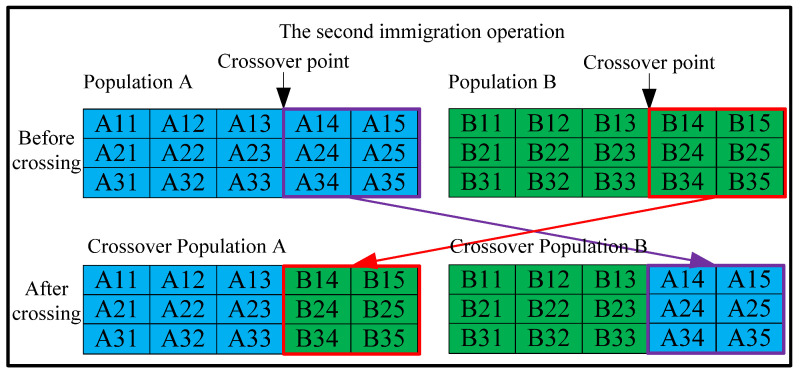
Design of the second immigration operator.

2. Immigration operator 2: When the best fitness values of the two populations do not change over several iterations, the immigration operator 2 is triggered, as depicted in Figure 4. Essentially, this operator involves swapping certain design variables of all individuals (except for the best individuals) between Population A and Population B. The purpose of designing this operator is to enable the algorithm to escape local optima.

## 5. Population Based on Reinforcement Learning Mechanism

When the solution space is vast, multiple local optima may exist, presenting challenges for algorithms that may become trapped in these local optima. To address this issue, Reference [20] proposes an innovative Nelder–Mead (NM) simplex method to solve the problem. Specifically, it involves a relocation operation on the solution, moving it away from local optimal regions to avoid premature convergence of the algorithm [20]. The key to preventing algorithms from falling into local optima is to enhance population diversity, a goal that Population A will focus on achieving. In GAs, the crossover operator significantly alters individual genes, making it a primary means of increasing population diversity. However, the relationship between CP and population diversity is ambiguous and time-variant, resembling a black box function that changes with algorithm iterations.

Empirical knowledge suggests that (1) a fixed CP is not optimal for optimization; (2) a low CP is not conducive to enhancing population diversity; and (3) a high CP tends towards random search. Based on these insights, some scholars designed adaptive formulas to control CP according to the population’s state [21]. However, these formulas might not necessarily lead to increased population diversity and could cause abrupt changes in CP in response to sudden changes in population state. Thus, the key to enhancing algorithm exploration lies in ensuring stability while reliably increasing population diversity. Reinforcement learning algorithms are well-suited to address these issues.

Reinforcement learning algorithms operate effectively in unknown or changing environments, as exemplified by the strategy network of the early version of AlphaGo (v. Lee) [22]. They allow agents to make decisions in the environment and adjust based on feedback, thus, obviating the need to elucidate the mapping relationship between CP and population diversity—essentially keeping the black box closed while still achieving desired objectives.

The next issue to address is how to make agents’ modifications to the CP more conducive to enhancing population diversity. The solution is straightforward: after each modification of CP by the agent, feed the standard deviation (STD) of population fitness values back to the agent as feedback. Since STD is the best reflection of population diversity, providing it to the agent will enable the agent to aim for enhancing population diversity while adjusting CP. This effectively tackles the challenge of time-varying black box functions that are difficult to definitively characterize.

### 5.1. Modular Design of Reinforcement Learning Mechanism

In the realm of reinforcement learning algorithms, Q-learning is one of the most widely applied methods [23,24,25]. It consists mainly of five components: agent, state space, action space, reward mechanism, and Q-table.

1. Agent: The agent is responsible for adjusting the CP within population A, taking over the role traditionally filled by adaptive formulas. When population A executes the crossover operator, the agent modifies the CP.
2. State Space: The state space is a collection of all possible values of CP, assuming Cmin and Cmax are the lower and upper bounds of CP, respectively, and StepS is the step size for changing CP. The size of the state space is 1 + (Cmax − Cmin)/StepS, meaning it is a set of [Cmin, Cmin + 1 × StepS, Cmin + 2 × StepS, …, Cmax]. The step size StepS of the state space can be determined by Equation (2), where SizeS represents the size of the state space.

(2) $$StepS=\left(Cmax-Cmin\right)/\left(SizeS-1\right)$$

Generally, if the problem to be solved can be simulated in less than 10 min for evaluating the EM scheme, then a larger value (e.g., above 20) can be set for SizeS. Conversely, if a single simulation of the problem is very time-consuming, then a smaller value (e.g., below 15) should be set for SizeS. Additionally, a rough surrogate model can be trained in advance with some collected samples, which can then be used to pre-train the agent, ensuring the algorithm starts with a relatively mature Q-table.

3. Action Space: The action space is a collection of all possible directions of change for the CP. As CP is a floating-point number, it can only change in three ways: increase, remain the same, or decrease. Thus, the size of the action space is 3, comprising the set: [Increase CP, Keep CP constant, Decrease CP].
4. Reward Mechanism: The reward mechanism provides feedback to the agent. After the agent modifies the CP, the environment provides a score to the agent through the reward mechanism, indicating the appropriateness of the agent’s decision-making. The reward is calculated using three indices from population A: the best fitness value (Bv), the average fitness value (Av), and the standard deviation of fitness values (Sv) as shown in Table 1. For instance, if Bv increases, Av remains constant, and Sv decreases, then the total reward for the agent is 5 + 0 − 3 = 2.

5. Q-table: The Q-table is a summary of the agent’s experience, updated with each reward the agent receives, making it progressively more intelligent. Typically, the action space constitutes the rows of the Q-table, while the state space forms its columns. Each cell in the Q-table represents the estimated reward (or Q-value) that the agent might receive for a certain action in a given state, as depicted in Table 2.

### 5.2. Updating Method of Q-Table

As agents modify the CP by consulting the action with the maximum value in the Q-table, the method of updating the Q-table significantly impacts the agent’s decision-making. Equation (3) is used for updating the Q-table. Q(s, a)^old^ is the estimated reward that the agent might receive for action (a) in state (s), obtained by consulting the Q-table. R represents the actual reward received from the environment after the agent takes action (a) in state (s). Once the agent executes action (a), the state of CP transitions from its original state (s) to state (s + 1). Max(Q(s + 1)) is the highest estimated reward the agent anticipates receiving by making decisions in state (s + 1), which can also be derived from the Q-table. α is the learning rate, and γ is the discount factor, both of which range from 0 to 1. Q(s, a)^new^ is the updated value of the Q-table cell.

(3) $$Q{\left(s,a\right)}^{\mathrm{new}}=\left(1-\alpha \right)\times Q{\left(s,a\right)}^{\mathrm{old}}+\alpha \times R+\alpha \times \gamma \times \mathrm{Max}\left(Q\left(s+1\right)\right)$$

The new value Q(s, a)^new^ is determined by three factors: Q(s, a)^old^, R, and Max(Q(s + 1)). Q(s, a)^old^ represents past experience, R represents current experience, and Max(Q(s + 1)) represents potential future experience. When α is small, the agent emphasizes past experience more. When α is large but γ is small, the agent prioritizes the current experience more. When both α and γ are large, the agent focuses more on potential future experience.

Equation (4) is employed to calculate the learning rate α, where αmin and αmax represent the lower and upper bounds of α, respectively, n denotes the iteration number, and h and d are coefficients to be determined. When the iteration number *n* is small, it implies that the algorithm is in its initial stages, with the Q-table nearly devoid of past experience. Consequently, a larger value of α is preferable at this stage to ensure that the agent places more emphasis on the current experience. Conversely, as n becomes large, it indicates that the algorithm has reached its later stages, and the agent has already accumulated a wealth of experience. At this point, it is beneficial to reduce the value of α to allow the agent to weigh past experiences more heavily.

(4) $$\alpha =\alpha min+\left(\alpha max-\alpha min\right)\times h\times e^{-d\times n}$$

Equation (5) is designed to calculate the discount factor γ, where n is the iteration number, t is an undetermined coefficient, and Bv is the current best fitness value. The discount factor γ plays a critical role in determining how much the agent values future rewards versus current rewards. When n is small, indicating the early stages of the algorithm, γ is decreased. This adjustment makes the algorithm prioritize the current rewards more significantly.

However, as per the implications of Equation (4), with an increase in n, the algorithm already places more emphasis on past experiences by decreasing α. Therefore, in the later stages of the algorithm, there is a sophisticated balance: the greatest emphasis is placed on past experiences, followed by future experiences, and lastly, the current experiences.

(5) $$\gamma =\gamma min+\left(\gamma max-\gamma min\right)\times t\times \frac{n}{maxn}\times Bv$$

### 5.3. The Process of Adjusting CP and Updating Q-Table

**Figure 5:**
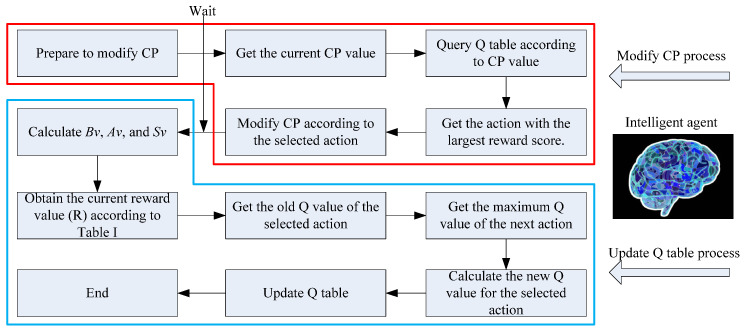
Process of modifying CP and updating Q-table.

When the algorithm executes the selection operator (as shown in the part enclosed by the red box on the left side of Figure 2), the agent enters the process of adjusting the CP (as shown in the part enclosed by the red box in Figure 5). The process is initiated with the agent obtaining the current value of CP to determine the state space it occupies. Subsequently, the agent consults the Q-table to identify the action that yields the highest reward. Following this identification, the agent modifies the CP based on the selected action. Finally, the agent enters a waiting state.

After the generation of the next population, the agent initiates the Q-table updating process (as illustrated in the part enclosed by the blue box in Figure 5). Once the Bv, Av, and Sv of the population are calculated, the agent computes the actual reward R received from the environment for the current action using Table 1. The agent then retrieves Q(s, a)^old^ and Max(Q(s + 1)) from the Q-table. At this point, the agent has acquired past, present, and future experiences and is ready to update the Q-table based on (3).

## 6. Design of Population Based on Leadership Dominance Mechanism

The key to efficiently optimizing LSS in EM design problems is to reduce the number of simulation iterations. If the exploitation of the algorithm is too weak, it will result in a greater number of iterations to find the optimal solution, and more iterations mean more simulation calls are needed. Consequently, a population with a leading dominance mechanism is designed (named Population B). This mechanism operates in the section framed by a green box on the right side of Figure 2.

### 6.1. Cross Method Based on Leadership Dominance Mechanism

**Figure 6:**
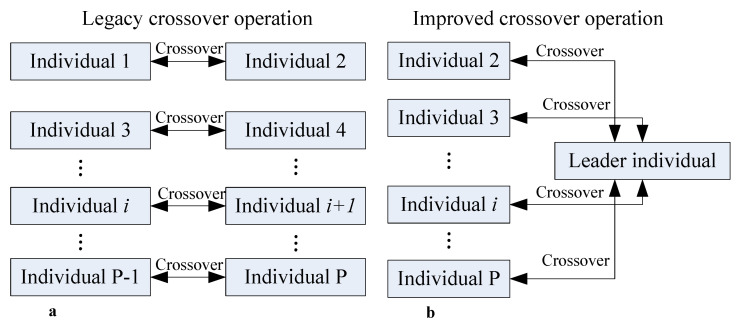
Difference between traditional crossover method and improved method. (**a**) Legacy crossover operation (**b**) Improved crossover operation.

The process of traditional crossover methods is depicted in Figure 6a. Initially, all individuals in the population are randomly paired two-by-two, and then each pair of individuals triggers a crossover operation with a certain probability. The disadvantage of this method is that the optimal individual in the population can only cross with other individuals at most once per iteration, preventing the rest of the population from fully learning the genetic pattern of the optimal individual. In contrast, the improved crossover method differs significantly from the traditional approach, as shown in Figure 6b. The detailed process is as follows: first, the individual with the highest fitness value in the current population is selected as the leader. Then, the remaining individuals cross over with the leader at a certain probability (CP). If an individual does not cross over with the leader, it will then cross over with another ordinary individual with a certain probability (Po) or not cross over at all with a probability (1-Po). As multiple individuals in the population may cross over with the leader due to this leading dominance mechanism, the genetic pattern of the leader is extensively mimicked, thereby rapidly enhancing the overall quality of the population.

### 6.2. Adjustment of Adaptive Crossover Probability

Equation (6) can be used to calculate CP, where CPmin and CPmax are the lower and upper bounds of CP, k is a coefficient to be determined, Fd is the fitness value of the leader, and f is the fitness value of the ordinary individual that needs to cross. The other parameters remain consistent with previous descriptions. When f < Av, indicating that the quality of the ordinary individual that needs to cross has not reached the average level of the population, CP will take its maximum value to encourage crossing with the leader. When f ≥ Av, if Sv is large, indicating good diversity within the population, CP should increase to allow more ordinary individuals to cross with the leader, enhancing the algorithm’s utilization capacity; otherwise, CP should decrease.

(6) $$CP=\left\{\begin{array}{l} CPmin+(CPmax-CPmin)\times k\times Sv\times \frac{F_{d}-f}{F_{d}-Av}\;\mathrm{if}\;f\geq Av\\ CPmax\;\;\;\;\;\;\;\;\;\;\;\;\;\;\;\mathrm{else} \end{array}\right.$$

## 7. Case Introduction

### 7.1. Introduction of Electromagnetic Supersurface

**Figure 7:**
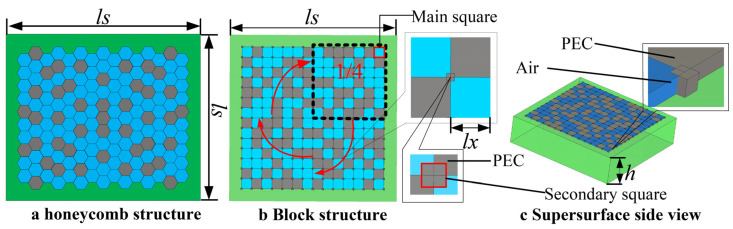
Structure of random lattice electromagnetic super-surface. (**a**) Honeycomb structure (**b**) Block structure (**c**) Supersurface side view.

To verify the effectiveness of the method proposed in this paper, we used the optimization of a random dot matrix EM metasurface as a case study, as shown in Figure 7. The optimization goal is to achieve as wide a zero-reflection phase bandwidth as possible within a specified frequency band (20–50 GHz). Therefore, the algorithm designed in this paper addresses the maximization optimization problem in the case study. There are two designs for the metasurface structure, as shown in Figure 7a,b. In general, to maintain the stability of the structure, the honeycomb structure in Figure 7a is preferred. However, due to the requirement of dual polarization for the metasurface in this case, the structure must satisfy 90° rotational symmetry. Clearly, Figure 7a does not meet this condition, so we choose the design in Figure 7b (i.e., first generating the lattice structure in the upper right corner and then rotating the structure 90° three times). To reinforce the structure, we added a small square at the junction of each main square in Figure 7b. The material of the main squares may be metal (PEC) or dielectric (air), while the material of the small squares must be metal. Since Figure 7b only shows information about the metasurface structure in the length and width dimensions, Figure 7c is used to display the information in the height dimension. Thus, Figure 7c is the complete side view of the metasurface, with the structure at the top being the same as the one shown in Figure 7b.

As can be seen from Figure 7b, the large squares within ls are not completely random, as three-fourths of the area are obtained by rotating one-fourth of the area. Nevertheless, the structure within the one-fourth region at the upper right corner still exhibits significant randomness. As mentioned in the introduction, a 7 × 7 structure has a solution space of 2^49^ = 562,949,953,421,312. Therefore, finding the optimal solution quickly within such a vast solution space remains a significant challenge.

Table 3 showcases the design variables of the EM metasurface, also illustrated in Figure 7. Among them, ls and h are set as fixed values; hence, they do not participate in the optimization. lx is the side length of the main square, and a is the ratio of the side length of the secondary square to the side length of the main square.

### 7.2. Design of Fitness Function

The metasurface is considered to have achieved a zero-reflection phase effect at a certain frequency point when the absolute value of the phase angle of S11 at that frequency is less than 90 degrees. The design process of the fitness function is as follows:

First, Equation (7) can calculate the phase angle of the metasurface at a certain frequency point, where S11(freq) can obtain S11 at frequency “freq”, and the functions Im() and Re() can obtain the imaginary and real parts of a complex number, respectively.

(7) Calphasefreq=arctanImS11freqReS11freq

Secondly, Equation (8) can determine whether the metasurface meets the conditions for zero-reflection-phase bandwidth at a certain frequency point. If the condition is satisfied, the function returns 1; otherwise, it returns 0.

(8) $$\mathrm{Meet}\left(freq\right)=\left\{\begin{array}{l} 1\;\;\mathrm{if}\;\left|\mathrm{Calphase}\left(freq\right)\right|<90{}^{\circ}\\ 0\;\;\mathrm{otherwise} \end{array}\right.$$

Finally, Equation (9) calculates the final fitness value, where stepf is the frequency step size. Essentially, the fitness value represents the proportion of frequency points satisfying the zero-reflection phase bandwidth condition in relation to the total cumulative frequency points.

(9) $$Fitnessvalue=\frac{\sum_{freq=20\mathrm{GHz}}^{freq=50\mathrm{GHz}}\mathrm{Meet}\left(freq\right)}{\left(\left(50-20\right)/stepf\right)+1}$$

## 8. Experimental Analysis

### 8.1. Experimental Method, Experimental Index, and Experimental Configuration

The experiment is divided into three parts: First, study the optimization results of IDPGA and traditional GA for a 7 × 7 random dot matrix EM metasurface. Second, investigate the optimization results of IDPGA and traditional GA for a 5 × 5 random dot matrix EM metasurface. Third, compare the performance of IDPGA with other advanced multi-population algorithms. The experiments are conducted using CST simulation software (CST Studio Suite 2022) on a Windows 10, 64-bit system with an AMD 3.4 GHz CPU and 128 GB RAM. In the first two parts of the experiment, since IDPGA is a dual population algorithm and traditional GA is a single population algorithm, the population size of traditional GA is set to double that of IDPGA to increase the fairness of the experiment (P = 40). The CP for traditional GA is set to 0.7, and the mutation probability is set to 0.03. The maximum number of iterations for all experiments is set to 100. In addition, the convergence criterion of the algorithm is as follows: when the optimal fitness value of the population remains unchanged for seven consecutive iterations, the algorithm is considered to have converged.

**Figure 8:**
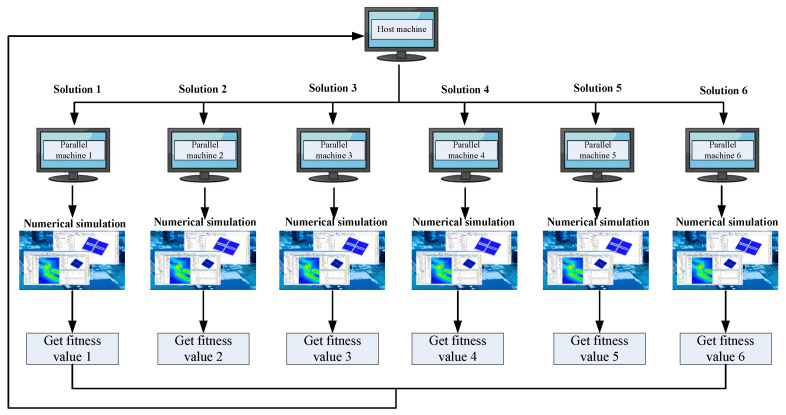
The flow of parallel computing.

In addition, since the evaluation order of solutions in the population does not affect the iterative results, we employed 10 computers for parallel computing to save solution evaluation time. This created two roles: the host and the parallel machines. The host is responsible for executing the algorithm proposed in this paper, while the parallel machines are responsible for invoking numerical simulations to evaluate the quality of the solutions. When the host generates a new population, all solutions in the population are simultaneously assigned to multiple parallel machines for computation. Once the fitness values of all solutions are calculated, the parallel machines return the fitness values to the host. The host then executes the operators designed in this paper and generates the next population, thus, completing the algorithmic process. As shown in Figure 8.

### 8.2. Optimization Results of 7 × 7 Random Lattice

**Figure 9:**
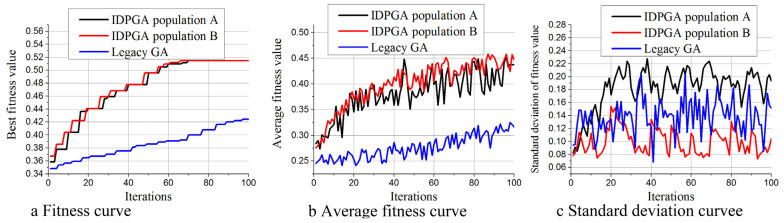
Performance comparison between IDPGA and traditional GA under 7 × 7 structure. (**a**) Fitness curve (**b**) Average fitness curve (**c**) Standard deviation curve.

Figure 9 illustrates the performance differences between IDPGA and traditional GA under a 7 × 7 structure. From Figure 9a, it is evident that the increase in Av of IDPGA is significantly faster than that of traditional GA. When IDPGA is iterated 66 times, it has already obtained a fitness value of 0.514, while the fitness value achieved by traditional GA is only 0.392. After undergoing 100 iterations, the fitness value of IDGPA remained unchanged, while the final fitness value obtained by traditional GA is 0.424. Figure 9b shows that the Av curve of traditional GA is always below that of IDPGA, indicating that IDPGA optimizes significantly faster than traditional GA. Figure 9c shows the changes in Sv for both methods. It is observed that Population A has the highest Sv, followed by traditional GA, and finally Population B. This is because Population A employs STD to continuously guide agents to enhance population diversity, resulting in the highest Sv for Population A. On the other hand, as a large number of individuals in Population B crossover with the leader, Population B tends to lose diversity more easily; hence, the lowest Sv for Population B. This aligns with the theoretical analysis presented earlier.

**Figure 10:**
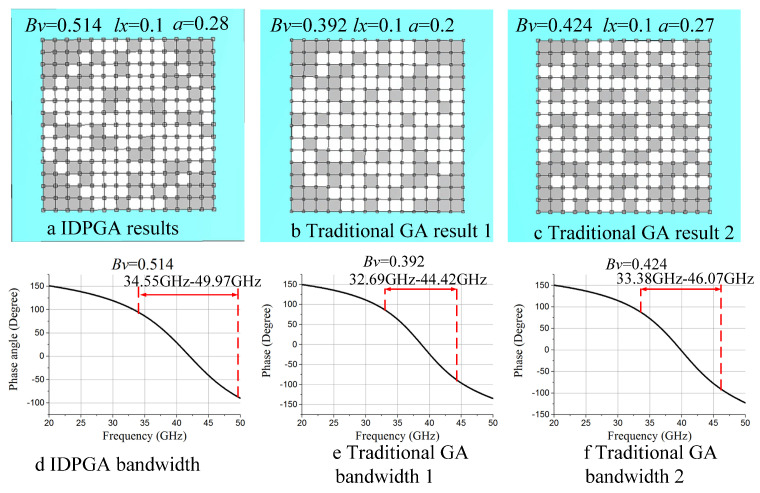
Optimization results of 7 × 7 structure by ID PGA and traditional GA. (**a**) IDPGA results (**b**) Traditional GA result 1 (**c**) Traditional GA result 2 (**d**) IDPGA bandwidth (**e**) Traditional GA bandwidth 1 (**f**) Traditional GA bandwidth 2.

Figure 10a,d displays the optimization results of IDPGA at iteration 66 (Bv = 0.514), which is also the final optimization result achieved by IDPGA. Figure 10b,e shows the optimization results of traditional GA at iteration 66 (Bv = 0.392). Figure 10c,f shows the final optimization results obtained by traditional GA (Bv = 0.424). It is evident that the EM metasurface structures corresponding to the three results vary significantly. In terms of zero-reflection-phase bandwidth, the optimal bandwidth achieved by IDPGA is 34.55 GHz to 49.97 GHz, while the bandwidth achieved by traditional GA is 33.38 GHz to 46.07 GHz. Therefore, IDPGA achieved a wider zero-reflection-phase bandwidth.

### 8.3. Optimization Results of 5 × 5 Random Lattice

**Figure 11:**
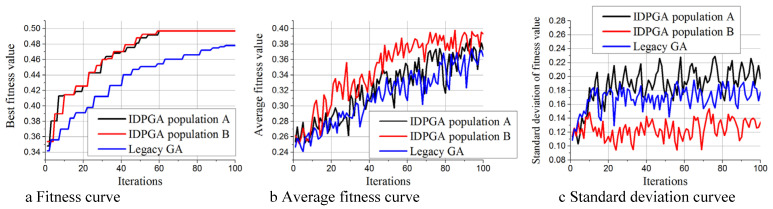
Performance comparison between IDPGA and traditional GA under 5 × 5 structure. (**a**) Fitness curve (**b**) Average fitness curve (**c**) Standard deviation curve.

Figure 11 shows the performance comparison of the two methods in optimizing a 5 × 5 structure. From Figure 11a, it is evident that IDPGA is about to converge at iteration 59, achieving a fitness value of 0.497, which is also the final optimization result of IDPGA. In contrast, traditional GA achieved a fitness value of 0.454 at iteration 59, and by iteration 100, it reached its final fitness value of 0.478. Thus, IDPGA still obtained better optimization results. Figure 11b indicates that although the growth rate of Av for IDPGA is still faster than that of traditional GA, the difference between the two is reduced compared to when optimizing the 7 × 7 structure. This suggests that the performance advantage of IDPGA diminishes somewhat when the solution space is reduced. Figure 11c shows that Population A’s Sv is still the highest, followed by traditional GA’s Sv, and finally Population B’s Sv. However, compared to optimizing the 7 × 7 structure, the difference in Sv between Population A and traditional GA has decreased. This indicates that when the solution space is reduced, the reinforcement learning mechanism of Population A’s ability to maintain population diversity is somewhat weakened.

**Figure 12:**
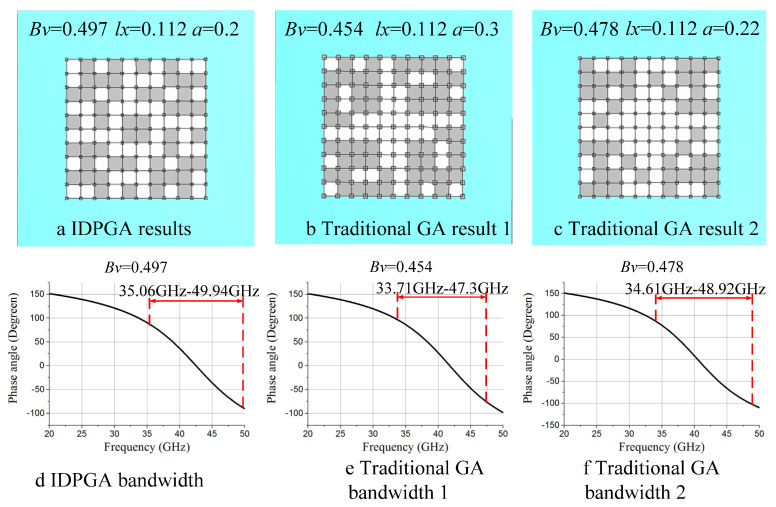
Optimization results of 5 × 5 structure by IDPGA and traditional GA. (**a**) IDPGA results (**b**) Traditional GA result 1 (**c**) Traditional GA result 2 (**d**) IDPGA bandwidth (**e**) Traditional GA bandwidth 1 (**f**) Traditional GA bandwidth 2.

Figure 12a,d shows the optimization results obtained by IDGPA (Bv = 0.497), Figure 12b,e shows the optimization results of traditional GA at iteration 59 (Bv = 0.454), and Figure 12c,f shows the final optimization results obtained by traditional GA (Bv = 0.478). The differences among the three optimized structures are still quite significant. The zero-reflection-phase bandwidth achieved by IDPGA is 35.06 GHz to 49.94 GHz, while the bandwidth achieved by traditional GA is 34.61 GHz to 48.92 GHz. Therefore, it can be concluded that IDPGA still achieves a wider zero-reflection-phase bandwidth under the 5 × 5 structure.

### 8.4. Performance Comparison Between IDPGA and Other Multi-Group Algorithms

To investigate the performance differences between IDPGA and other algorithms, several advanced multi-population methods are selected for comparison. These include the Shuffled Leapfrog Algorithm (SLFA) [26], the Seeker Algorithm (SA) [27], and the Multi-Population Differential Evolution Algorithm (MPDEA) [28], with parameters referenced from the original publications. As MPDEA is a multi-objective algorithm, its non-dominated sorting module is replaced with a fitness calculation module for better comparability, and its mutation strategy is DE/current-to-best/1. In SLFA, the concept of “stone” is analogous to populations in IDPGA, so the number of “stone” is set to 2. SA employs a Gaussian distribution function to measure search step size. To enhance comparability, all populations in SA are merged for Sv calculation. The rest of the parameters for all methods are consistent with previous experiments to maintain comparability.

**Figure 13:**
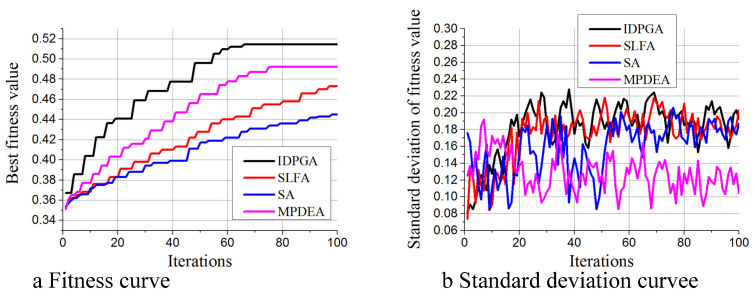
Performance differences in each multi-population algorithm. (**a**) Fitness curve (**b**) Standard deviation curve.

Figure 13a shows the Bv variation curves of all methods, indicating that IDPGA has the fastest growth rate, followed by MPDEA, then SLA, and lastly SA. Figure 13b displays the Sv variations, revealing that IDPGA and SLFA have the largest Sv, closely followed by SA, with MPDEA being the least. Thus, IDPGA’s ability to maintain population diversity is comparable to that of SLFA. The lower diversity in MPDEA is attributed to its greedy strategy for selecting the next generation, which reduces randomness and, consequently, population diversity.

**Figure 14:**
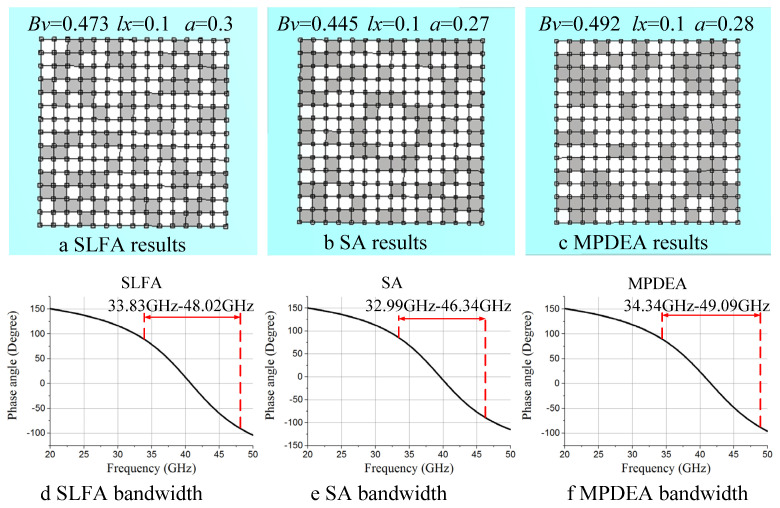
Optimization results obtained by different multi-population algorithms. (**a**) SLFA results (**b**) SA results (**c**) MPDEA results. (**d**) SLFA bandwidth. (**e**) SA bandwidth. (**f**) MPDEA bandwidth.

Figure 14 illustrates the optimization results of the 7 × 7 structure for SLFA, SA, and MPDEA. There are significant differences in the structures optimized by the four methods. MPDEA achieved a bandwidth of 34.34–49.09 GHz, relatively close to IDPGA’s, suggesting that despite a lower Bv compared with IDPGA, MPDEA is still suitable for solving LSS EM design problems. SLFA achieved a bandwidth of 33.83–48.02 GHz, and SA achieved 32.99–46.34 GHz, both inferior to IDPGA and MPDEA. Therefore, it can be concluded that while both IDPGA and MPDEA demonstrate good performance, IDPGA still surpasses MPDEA.

## 9. Conclusions

This paper proposes a dual-population algorithm to address the large solution space problem in electromagnetic optimization design. The first population employs reinforcement learning to adjust the crossover probability, thereby enhancing the global optimization capability of the algorithm. The second population adopts a leader-dominance mechanism to ensure that the current optimal solution is effectively imitated, thereby improving the convergence speed of the algorithm. The two populations integrate their advantages through an immigration operator, enabling rapid search for the optimal solution in a large solution space. An experiment on electromagnetic metasurface materials demonstrates that the proposed algorithm can find the optimal solution with fewer iterations, significantly reducing the dependence on electromagnetic numerical simulations. The proposed algorithm outperforms legacy GA and other similar algorithms in both global optimization capability and convergence speed. Since the proposed algorithm belongs to the class of heuristic search algorithms, it can efficiently solve most NP problems in theoretical informatics. Therefore, its application is not limited to the field of electromagnetics. Other engineering optimization design problems with high computational costs, such as aerodynamic drag minimization of aircraft wings, bridge structure optimization, robot obstacle avoidance path optimization, and power dispatch optimization, can also be efficiently solved using this method. This approach effectively reduces the computational time cost of optimization design while improving the optimization speed.

## Figures and Tables

**Table 1:** The design of the reward mechanism.

Indicator	Bv	Av	Sv
Increase	+5	+1	+3
Unchanged	+0	+0	+0
Decrease	−5	−1	−3

**Table 2:** The structure of the Q-table.

	CP Is Increased	CP Remains Unchanged	CP Is Decreased
CP = Cmin	Q(1, 1)	Q(1, 2)	Q(1, 3)
……	Q(m, 1)	Q(m, 2)	Q(m, 3)
……	Q(n, 1)	Q(n, 2)	Q(n, 3)
CP = Cmax	Q(k, 1)	Q(k, 2)	Q(k, 3)

**Table 3:** The design variables of the EM hypersurface.

Name	Range/mm	Description
ls	1.92	Random lattice side length
h	0.48	Random lattice height
*lx*	0.08~0.16	Main block side length
*a*	0.1~0.4	Side length ratio

## Data Availability

The original contributions presented in the study are included in the article; further inquiries can be directed to the corresponding author.

## References

[1] Shao Z.J., Qiu L.F., Zhang Y.P. (2020). Design of Wideband Differentially Fed Multilayer Stacked Patch Antennas Based on Bat Algorithm. IEEE Antennas Wirel. Propag. Lett..

[2] Zhou J.Z., Yang Z.B., Zhang Z.Y. (2021). A Trust-Region Parallel Bayesian Optimization Method for Simulation-Driven Antenna Design. IEEE Trans. Antennas Propag..

[3] Bilal R.M., Naveed M.A. (2021). A comment: A set square design metamaterial absorber for X-band applications. J. Electromagn. Waves Appl..

[4] Sarmah K., Goswami S., Baruah S. (2020). Surrogate Model Assisted Design of CSRR Structure using Genetic Algorithm for Microstrip Antenna Application. Radioengineering.

[5] Rao K.N., Meshram V., Suresh H.N. (2020). Synthesis of ultra wideband tightly coupled array with RFSS by using particle swarm optimization algorithm. Microw. Opt. Technol. Lett..

[6] Jian R.L., Chen Y.Y., Chen T.H. (2019). Multi-Parameters Unified-Optimization for Millimeter Wave Microstrip Antenna Based on ICACO. IEEE Access.

[7] Hu C.E., Zeng S.Y., Gao S. (2019). A Robust Technique Without Additional Computational Cost in Evolutionary Antenna Optimization. IEEE Trans. Antennas Propag..

[8] Fu K., Cai X., Yuan B., Yang Y., Yao X. (2022). An efficient surrogate assisted particle swarm optimization for antenna synthesis. IEEE Trans. Antennas Propag..

[9] Zhang Z., Chen H.C., Cheng Q.S. (2021). Surrogate-Assisted Quasi-Newton Enhanced Global Optimization of Antennas Based on a Heuristic Hypersphere Sampling. IEEE Trans. Antennas Propag..

[10] Qian J.C., Cheng Y.S., Zhang J.L. (2021). Optimization design of metamaterial vibration isolator with honeycomb structure based on multi-fidelity surrogate model. Struct. Multidiscip. Optim..

[11] Ustun D., Toktas F. (2021). Surrogate-based computational analysis and design for H-shaped microstrip antenna. J. Electromagn. Waves Appl..

[12] Liu B., Koziel S., Ali N. (2017). SADEA-II: A generalized method for efficient global optimization of antenna design. J. Comput. Des. Eng..

[13] Liu B., Akinsolu M.O., Song C.Y., Hua Q., Xu Q., Huang Y., Imran M.A. (2021). An Efficient Method for Complex Antenna Design Based on a Self Adaptive Surrogate Model-Assisted Optimization Technique. IEEEE Trans. Antennas Propag..

[14] Liu Y.S., Liu B., Hua Q. (2022). An Efficient Method for Antenna Design Based on a Self-Adaptive Bayesian Neural Network-Assisted Global Optimization Technique. IEEE Trans. Antennas Propag..

[15] Shi S., Zhang X.L., Wang Y.J. (2019). Prediction of the RNA Secondary Structure Using a Multi-Population Assisted Quantum Genetic Algorithm. Hum. Hered..

[16] Wang Y.H., Liu S.F., Yu H.D. (2020). On-demand optimize design of sound-absorbing porous material based on multi-population genetic algorithm. e-Polymers.

[17] Dang X.Y., Gong D.W., Liu H. (2022). Enhancement of Mutation Testing via Fuzzy Clustering and Multi-Population Genetic Algorithm. IEEE Trans. Softw. Eng..

[18] Zhao G.H., Shen W.H., Wu M.L. (2009). Ultra-Wideband Antenna Design Using FDTD and Double Population Genetic Algorithm. Microw. Opt. Technol. Lett..

[19] Shang F., Feng T.Y., Wang W.S. (2008). A new method for optimum design of Yagi-UDA antenna. J. China Acad. Electron. Inf. Technol..

[20] Steffen V. (2022). Particle Swarm Optimization with a Simplex Strategy to Avoid Getting Stuck on Local Optimum. AI Comput. Sci. Robot. Technol..

[21] Zhai L.Z., Feng S.H. (2022). A novel evacuation path planning method based on improved genetic algorithm. J. Intell. Fuzzy Syst..

[22] Silver D., Huang A., Maddison C.J., Guez A., Sifre L., van den Driessche G., Schrittwieser J., Antonoglou I., Panneershelvam V., Lanctot M. (2016). Mastering the game of Go with deep neural networks and tree search. Nature.

[23] Clifton J., Laber E. (2020). Q-Learning: Theory and Applications. Annu. Rev. Stat. Its Appl..

[24] Low E.S., Cheah K.C. (2019). Solving the optimal path planning of a mobile robot using improved Q-learning. Robot. Auton. Syst..

[25] Low E.S., Ong P., Low C.Y. (2021). Mobile Robot Path Planning using Q-Learning with Guided Distance and Moving Target Concept. Int. J. Integr. Eng..

[26] Kaveh A., Talatahari S., Khodadadi N. (2019). The Hybrid Invasive Weed Optimization-Shuffled Frog-leaping Algorithm Applied to Optimal Design of Frame Structures. Period. Polytech.-Civ. Eng..

[27] Duan S.M., Luo H.L., Liu H.P. (2022). An Elastic Collision Seeker Optimization Algorithm for Optimization Constrained Engineering Problems. Math. Probl. Eng..

[28] Wang J.H., Zhang W.W., Zhang J. (2016). Cooperative Differential Evolution With Multiple Populations for Multiobjective Optimization. IEEE Trans. Cybern..

